# Biomechanical Design Optimization of Distal Humerus Fracture Plates: A Review

**DOI:** 10.1155/2024/6015794

**Published:** 2024-06-27

**Authors:** Radovan Zdero, Pawel Brzozowski, Emil H. Schemitsch

**Affiliations:** ^1^ Orthopaedic Biomechanics Lab Victoria Hospital, London, Ontario, Canada; ^2^ Division of Orthopaedic Surgery Western University, London, Ontario, Canada

**Keywords:** biomechanics, distal humerus, fracture, optimization, plates, review

## Abstract

The goal of this article was to review studies on distal humerus fracture plates (DHFPs) to understand the biomechanical influence of systematically changing the plate or screw variables. The problem is that DHFPs are commonly used surgically, although complications can still occur, and it is unclear if implant configurations are always optimized using biomechanical criteria. A systematic search of the PubMed database was conducted to identify English-language biomechanical optimization studies of DHFPs that parametrically altered plate and/or screw variables to analyze their influence on engineering performance. Intraarticular and extraarticular fracture (EAF) data were separated and organized under commonly used biomechanical outcome metrics. The results identified 52 eligible DHFP studies, which evaluated various plate and screw variables. The most common plate variables evaluated were geometry, hole type, number, and position. Fewer studies assessed screw variables, with number and angle being the most common. However, no studies examined nonmetallic materials for plates or screws, which may be of interest in future research. Also, articles used various combinations of biomechanical outcome metrics, such as interfragmentary fracture motion, bone, plate, or screw stress, number of loading cycles to failure, and overall stiffness (Os) or failure strength (Fs). However, no study evaluated the bone stress under the plate to examine bone “stress shielding,” which may impact bone health clinically. Surgeons treating intraarticular and extraarticular distal humerus fractures should seriously consider two precontoured, long, thick, locked, and parallel plates that are secured by long, thick, and plate-to-plate screws that are located at staggered levels along the proximal parts of the plates, as well as an extra transfracture plate screw. Also, research engineers could improve new studies by perusing recommendations in future work (e.g., studying alternative nonmetallic materials or “stress shielding”), clinical ramifications (e.g., benefits of locked plates), and study quality (e.g., experimental validation of computational studies).

## 1. Introduction

Distal humerus fractures are usually due to a fall [1]. The Orthopaedic Trauma Association (OTA) categorizes them as Type 13 with various intraarticular, partial articular, and extraarticular subtypes [2]. Distal humerus fracture plates (DHFPs) are the primary surgical treatment for most of these injuries, but postoperative complications can occur in 53% of cases (e.g., malunion, nonunion, infection, and nerve injury), and reoperations may happen in 21% of cases [3, 4]. This raises a question: Is the specific DHFP configuration being implanted due to commercial availability, economic cost, regulatory policy, surgical experience, patient-based factors, clinical evidence, biomechanical criteria, etc. [5, 6]?

A starting point, therefore, for designing, analyzing, or utilizing DHFPs may be to employ biomechanical optimization criteria (BOC) [7]. BOC1 asserts that axial (i.e., perpendicular) motion relative to the fracture is moderate (i.e., within 0.2–1 mm), whereas shear (i.e., parallel) motion relative to the fracture occurs such that the shear/axial fracture motion ratio is low (i.e., < 1.6), to potentially encourage early callus formation [8–10]. BOC2 asserts that bone and plate stress (i.e., MPa units) stays below the ultimate tensile stress of the constituent materials to lessen the mechanical failure risk [11, 12]. BOC3 asserts that bone stress (i.e., MPa units) at the bone-plate interface equals or exceeds the stress at corresponding locations on an intact bone or bone-plate “control” in order to minimize “stress shielding” [13, 14]. BOC4 asserts that the bone-plate construct undergoes 150,000 or more load cycles, which corresponds to 2 months or more of clinical use to allow time for fracture healing [15]. However, it is unclear how often these BOCs are used for studying the engineering performance of DHFPs.

Accordingly, there are reasons why a survey on biomechanical design optimization of DHFPs may be necessary. First off, biomechanical reports on DHFPs often only measure or compute construct stiffness (i.e., N/mm or Nm/deg units) and/or failure strength (Fs) (i.e., N or Nm units) [16–18]; however, these outcomes may not be sufficient to understand and improve DHFP performance according to the BOCs above. Moreover, biomechanical reports on the plating of proximal [19–21] or midshaft [22–24] humerus fractures may not always be completely applicable to distal humerus fractures because of different geometries, bone densities, and stress distributions [25–28]. Furthermore, the few prior reviews that addressed DHFP biomechanics only considered parallel versus perpendicular double plating (i.e., the only variable was plate position) [29], or they only had brief sections on biomechanics with few references and cursory discussions on optimizing plate or screw variables [30, 31].

Therefore, this review comprehensively discusses articles that optimized or characterized the engineering performance of DHFPs by systematically permutating plate or screw variables. This information can assist engineers and surgeons in designing, analyzing, and utilizing DHFPs.

## 2. Methods

The PubMed database was searched for articles via the terms “biomechanics” plus “distal humerus” plus “fracture” or “plate”. Inclusion criteria were (i) biomechanical optimization studies that parametrically analyzed DHFPs by altering plate and/or screw variables instead of comparing them against or combining them with other implants (e.g., nails, pins, and wires), (ii) intraarticular fractures (IAFs) or extraarticular fractures (EAFs), (iii) any publication date, and (iv) English-language studies. IAFs and EAFs are often surgically plated in identical or similar ways, but they are treated separately here to highlight potential differences. Every plate or screw variable mentioned below is discussed using all the studies that examined that variable, whether they involved a few or numerous studies. Relative biomechanical changes caused by permutating plate or screw variables are primarily given below since they are more important than absolute numerical data. Only mean or median data are considered in summarizing the findings below, but statistical “*p*” values are not included because studies often lack enough specimens per group to realize all statistical differences. Only one of the current authors extracted the numerical data from the reviewed papers to determine the influence of plate and/or screw variables, which may have introduced some biases in data interpretation, although it did provide consistency in presenting the findings below. Specifically, to standardize data extraction and minimize data bias, the following guidelines were used. (i) All numerical values were extracted from the main text of each paper when available; otherwise, a scaled ruler was used to estimate the numerical values from the figures. (ii) IAF or EAF data were separated. (iii) Data was collected systematically: first, all data pertaining to interfragmentary fracture motion (i.e., BOC1) was searched; second, bone, plate, or screw stress (i.e., BOC2); third, bone stress under the plate (i.e., BOC3); fourth, the number of loading cycles to failure (i.e., BOC4); fifth, overall stiffness (Os) or Fs; and finally, any other study data was searched to ensure no information was missed. (iv) Each paper was reviewed at least twice to ensure all plate or screw permutations investigated were recorded.

## 3. Results

### 3.1. Study Characteristics

**Figure 1:**
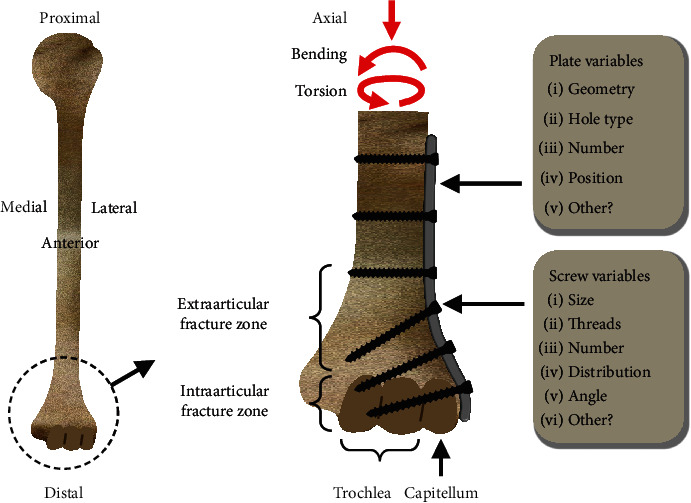
Typical DHFP variables. The OTA classifies distal humerus fractures (not shown) as Type 13 with various intraarticular, partial articular, and extraarticular subtypes. Red arrows indicate axial, bending, and torsion loads commonly experienced by the distal humerus.

**Figure 2:**
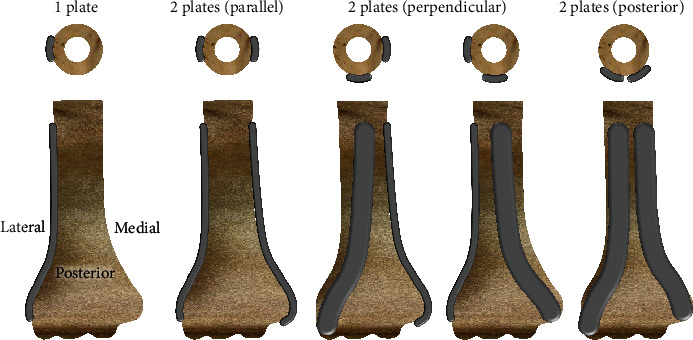
Typical DHFP positions. Top views of cross-sections only show plate locations on the humerus shaft. Parallel plates are sometimes called a 180° configuration. Perpendicular plates are sometimes called a 90° configuration. Fractures and screws are not shown.

The PubMed search initially uncovered 390 studies. After using the inclusion criteria, only 52 studies (i.e., 13%) were eligible. Plate variables were geometry, hole type (i.e., threaded locked vs. nonthreaded nonlocked), number, and position (Figures 1 and 2). Screw variables were size, threads, number, distribution, and angle (Figure 1). Outcome metrics for IAFs [32–58] and EAFs [16–18, 59–80] could be fracture motion (i.e., BOC1); bone, plate, or screw stress (i.e., BOC2); bone stress under the plate (i.e., BOC3); and “fatigue life,” defined as the number of loading cycles needed for failure (i.e., BOC4), as well as Os and Fs (Tables 1, 2, and 3).

### 3.2. Plate Geometry

For IAFs, a single posterior-lateral plate twice the length but half the width of a triangular antiglide plate had lower axial stiffness but higher axial strength when fixing a simple capitellum fracture [33], but it had greater axial stiffness and strength for a complex capitellum fracture [34]. Thicker versus thinner double plates had smaller axial stiffness but less fracture motion, a longer bending fatigue life, and greater bending stiffness and strength [52]. A single Y-plate versus double plates with standard shapes in parallel or posterior arrangements had higher axial stiffness but more fracture motion and lower axial strength, bending stiffness, and bending strength [47]. A single Y-plate designed to match bone geometry had less bone, plate, and screw stress in axial, bending, and torsional loading versus a single traditional Y-plate [58].

For EAFs, a single long curved J-plate had greater bending stiffness as well as more torsional stiffness and strength versus a single straight short plate [77], but it only had higher stiffness or strength in half the loading modes versus a single wider upside-down proximal humerus plate repurposed for the distal humerus [71]. Longer versus shorter double plates exhibited less fracture motion [80], reduced bone and plate stress [80], and higher stiffness and/or strength in various loading modes [64, 80]. A single Y-plate versus double plates with standard shapes in parallel or perpendicular configurations had more fracture motion [59, 75], higher bone and plate stress [75], shorter axial and bending fatigue life [65], or lower stiffness in most loading modes [59, 65, 75], but one study showed the reverse for fracture motion and stiffness in bending [75].

Key Concept: Plates that are longer, thicker, or contoured to better match bone geometry increase construct rigidity. Also, double plates with standard shapes perform better than a single plate with a Y shape.

### 3.3. Plate Hole Type

Double-locked versus nonlocked plates for IAFs usually had more axial stiffness, bending stiffness, axial strength, and/or axial fatigue life, as well as less fracture motion, in high and low-density bone [38, 44, 52], but there were some exceptions in high-density bone [38]. Single- or double-locked versus nonlocked plates for EAFs sometimes had higher or lower fracture motion, stiffness, and/or strength during various loading modes, possibly due to variations in comminution and study protocol [59, 61, 63, 69, 70].

Key Concept: Locked plates generally create more, or equally, rigid constructs due to the interdigitation of screw head threads with plate hole threads, unlike nonlocked plates.

### 3.4. Plate Number

Double plating without augmentation screws versus single plating plus a transfracture lag screw for IAFs showed greater axial, bending, and torsional stiffness, as well as more bending strength, but may or may not achieve higher axial strength [48, 57]. Double versus single plating of EAFs almost always exhibited less fracture motion, lower stress (i.e., bone and plate), higher stiffness (i.e., axial, bending, and torsion), and/or greater strength (i.e., bending and torsion) [16, 18, 62, 76–79]. One EAF study showed that double versus single plates had less axial strength, possibly due to unequal bone quality [18]. Triple-plate versus various double-plate constructs for EAFs almost always had greater bending and torsional stiffness [66].

Key Concept: More versus fewer plates usually generate more rigidity due to additional buttressing by extra plates.

### 3.5. Plate Position

For IAFs, parallel versus perpendicular double plating had less fracture motion [41–43, 54, 55]; smaller bone, plate, and/or screw stress [35, 40]; longer fatigue life [54]; higher axial, bending, and/or torsional stiffness [32, 35, 36, 40, 42, 43, 46, 49–51, 54–57]; and greater axial, bending, and/or torsional strength [32, 43, 46, 49, 51, 56, 57]. However, some comparisons showed that parallel versus perpendicular double plating had lower or equivalent stiffness and strength, leading to more fracture motion and plate stress, likely due to different study protocols [32, 36, 40, 43, 45, 54]. Moreover, one investigation reported that posterior versus parallel double plating had less axial stiffness and more bone stress [35]. In contrast, a different study showed that posterior versus parallel double plating exhibited less fracture motion, higher axial and bending stiffness, and greater bending strength but lower axial strength [47]. Another paper demonstrated that novel perpendicular double plating (i.e., anterior–medial plus anterior–lateral) versus traditional perpendicular double plating (i.e., medial plus posterior-lateral) resulted in less plate stress; lower bone and screw stress in most cases; and higher axial, bending, and torsional stiffness [53].

For EAFs, parallel versus perpendicular double plating exhibited less fracture motion, smaller bone or implant stress, longer fatigue life, greater stiffness, and/or higher strength [17, 60, 61, 67–69, 75, 76], although one study showed the reverse for bending and torsional stiffness [66]. Several studies also showed that parallel and/or perpendicular versus posterior double plating had less fracture motion, smaller plate stress, greater stiffness, and/or higher strength in most cases of axial, bending, or torsional loading [60, 70]. A few investigations reported that a single posterior-lateral plate had more axial, bending, and torsional stiffness than a single lateral plate [16], but it had lower stiffness and strength in most cases versus a single anterior–lateral plate [73].

Key Concept: Parallel double plating is more rigid, possibly due to its symmetrical mechanical support and longer “lever arm” distance to the humerus long axis versus perpendicular double plating, although some studies show the opposite. However, more work could be done on optimizing the position of perpendicular double plates, posterior double plates, and single plates.

### 3.6. Screw Size

Double plating of IAFs using larger versus smaller diameter screws produced more axial stiffness but less anterior–posterior bending stiffness [38]. Double plates, whereby one plate had more bicortical (i.e., longer) than unicortical (i.e., shorter) screws to repair EAFs, had more axial stiffness but less anterior–posterior bending stiffness, versus double plates, in which one plate used more unicortical than bicortical screws [72]. The following reasons may explain the data. Axial load acted perpendicularly to each screw axis, so thicker screws had larger cross-sectional areas that better resisted motion and longer screws provided more buttressing length; thus, axial stiffness increased. Conversely, anterior–posterior bending load produced circumferential rotation around each screw axis, so thicker, and longer screws replaced more bone that allowed more rotation; thus, anterior–posterior bending stiffness decreased.

Key Concept: Thicker and longer screws increase construct rigidity during axial loads, but not during anterior–posterior bending loads.

### 3.7. Screw Threads

Double plating that replaced several regular screws proximally and distally with plate-to-plate bolts (i.e., shallower and closer threads secured to the plate via nuts) had higher axial stiffness and strength for IAFs versus double plating using all regular screws [46].

Key Concept: Plate-to-plate bolts create a single implant structure for more compression of bone fragments, while there are smaller stress risers around the shallower and closer threads of bolts versus regular screws.

### 3.8. Screw Number

A single plate plus a transfracture lag screw for IAFs experienced more axial and bending strength after adding a transcondylar (i.e., not transfracture) screw through the plate [48], but double plating may or may not have benefited from this strategy for axial stiffness and fracture motion [37]. Double plating of IAFs achieved greater axial stiffness, bending stiffness, and axial strength, but not axial fatigue life, after inserting an extra plate screw across the fracture [38].

Key Concept: Adding a transcondylar (i.e., not transfracture) plate screw enhances the rigidity of single, but not necessarily double, plate constructs, although an extra transfracture plate screw boosts double plate rigidity.

### 3.9. Screw Distribution

Double plating of EAFs experienced higher bending strength when the most proximal screw in each plate was at staggered locations to reduce stress concentrations versus screws placed at the same level [64]. This was true whether or not the plates had the same or different lengths.

Key Concept: Proximal screw distributions at staggered levels improve construct rigidity, although more work is needed.

### 3.10. Screw Angle

Double plating of IAFs using only fixed-angle transverse screws generated lower plate stress and less fracture motion but more bone stress for most loading modes versus double plating that used variable-angle screws on the lateral side [39]. This conflicts with another study on double plating of IAFs, which demonstrated that fixed-angle transverse screws produced lower axial stiffness and strength when using parallel plates, but the trend was reversed for perpendicular plates [51]. Double plating of EAFs using only fixed-angle transverse screws provided higher axial stiffness and force at smaller, but not at larger, construct displacements versus double plating using only variable-angle screws [74].

Key Concept: It is unclear if fixed-angle or variable-angle screws create more rigid constructs, thereby warranting more research.

### 3.11. Summary of Findings

The “key concept” statements above for each plate or screw variable could be combined to suggest a potentially optimal DHFP configuration (Tables 1 and 2). As such, a distal humerus IAF or EAF might ideally be fixed using two precontoured, long, thick, locked, parallel metal plates. They would be affixed to the bone using long, thick metal screws that extend from plate to plate, but the screws should be located at staggered levels along the proximal sections of the plates. This would be supplemented by an extra transfracture plate screw made from metal. However, potential nonmetallic materials for plates or screws were not evaluated by any studies, while screw angles seemed to have little influence, so no recommendations can be made on these items.

The full range of outcome metric data could give insights into the performance of the reviewed DHFPs (Table 3). First, the wide range of data showed that performance depended on the DHFP's plate and/or screw configuration. Second, there were overlapping data for IAF versus EAF studies that both reported the same outcome metrics (nine of 10 cases), suggesting similar success in treating both types of injuries. Third, several outcome metrics were not examined by DHFP studies (eight of 18 cases), illustrating the lack of widely-used standardized modeling and testing protocols. Fourth, axial interfragmentary motion (IFM) for many DHFPs overlapped with the 0.2–1 mm range suggested for early healing, but two studies had shear IFM/axial IFM ratios that sometimes [41] or always [43] exceeded 1.6, thereby compromising early healing (i.e., BOC1) [8–10]. Fifth, bone, plate, and/or screw peak stresses (*σ*) for most DHFPs were below the ultimate tensile strengths of cortical bone (50–146 MPa), steel (465–950 MPa), and/or titanium (960–970 MPa) (i.e., BOC2) [11, 12], thereby decreasing failure risk. Sixth, DHFPs were never assessed for bone stress under the plate (*σ*_BUP_) to evaluate bone “stress shielding” risk (i.e., BOC3) [13, 14], which could cause bone density loss and plate loosening. Seventh, DHFPs never reached 150,000 loading cycles which is sometimes proposed to allow enough time for fracture healing (i.e., BOC4) [15]. Eighth, Os and Fs were used for relative comparisons between different DHFPs, but they were rarely compared to an intact “control” humerus as a baseline, so their value as outcome metrics for design optimization was unclear.

It should be emphasized that the above-recommended DHFP configuration, as well as the range of data from the reviewed studies, can be influenced by factors that are beyond the control of engineers or surgeons in a real clinical context, that were not always well documented in the above studies, and/or that were not always assessed by investigators, such as implant geometry and material, bone geometry and density, load type and magnitude, and fracture type and size.

## 4. Discussion

### 4.1. Plate and Screw Factors

The most commonly studied DHFP variable was plate position (30 studies), while fewer papers considered geometry (12 studies), number (10 studies), and hole type (eight studies). The most studied screw variables were number (three studies) and angle (three studies), while fewer papers examined size (two studies), threads (one study), or distribution (one study). Alternative nonmetallic materials (e.g., fiber-reinforced polymers) for plates or screws were never examined, yet plates made from such materials for other long bones permit better control of fracture motion, plate stress, stress shielding, etc. [81–83]. Also, screw threads and screw distribution received little attention, unlike plating of the proximal humerus [84] or distal femur [7]. Thus, more DHFP research is needed on alternative materials and particular screw variables. Furthermore, a few studies only partly succeeded in changing one plate or screw variable at a time, since there were confounding variables (e.g., one locked plate vs. two nonlocked plates [78]; one Y-plate vs. two plates with a standard shape [47, 59, 65, 75]). As such, the influence of a particular variable was sometimes unclear, especially while conducting parametric studies using commercial DHFPs which have many different designs (Table 4) [85–87]. This could lead to misleading recommendations to engineers and surgeons about the optimal implant configuration. Thus, future DHFP investigators could make 3D-printed custom plates [88] or use finite element models (FEMs) [89] to systematically alter each variable.

### 4.2. Patient Factors

Several patient factors may affect DHFP performance that are beyond the control of engineers or surgeons. Firstly, simulated IAFs (e.g., well-defined smooth cuts [43]) and EAFs (e.g., transverse fracture gaps [78]) in the reviewed studies may not fully represent the size, shape, direction, or number of fracture lines seen clinically [2]. Thus, future DHFP researchers might develop a standardized methodology to create more realistic fractures. Secondly, the reviewed studies usually replicated normal (e.g., [32]) or osteoporotic (e.g., [75]) humeri, but these can generate different results because normal cortical bone has a 52% larger elastic modulus [90] and a 23% greater ultimate tensile stress [91] versus osteoporotic cortical bone. Thus, future DHFP work in the same study could compare normal versus osteoporotic humeri, as done by three studies [52, 59, 61]. Thirdly, the reviewed investigations utilized axial (42 studies), bending (38 studies), and/or torsional (25 studies) loading, but fewer papers employed all three loading modes (15 studies). However, forces at the shoulder and elbow joints [92, 93], as well as bone and implant stresses [67, 94], can vary depending on humerus orientation during various tasks [95, 96]. Thus, future DHFP investigations should use all three loading modes.

### 4.3. Study Quality

There were 28 of 42 reviewed experimental papers that used animal or human humeri to bolster confidence in results; however, the artificial humeri used by the other studies generally exhibit realistic mechanical behavior [97, 98]. Thus, future DHFP researchers should consider using cadaveric humeri or, alternatively, artificial humeri that have been thoroughly validated.

Moreover, two of the 10 reviewed FEM investigations also performed experiments for validation, thereby raising confidence in the data; however, the other FEM studies did employ known prior experimental data (e.g., elastic modulus, Poisson's ratio, and bone–metal friction coefficient) to develop their models [89]. Thus, future DHFP researchers could improve FEM reliability by comparing results to their own experiments or from previous publications.

Also, the reviewed studies reported outcomes that could be compared to BOC1 (17 studies), BOC2 (11 studies), BOC3 (zero studies), and/or BOC4 (six studies), whereas 27 studies only provided Os and/or Fs, which are too imprecise for biomechanical optimization. Thus, future DHFP researchers could ideally employ all four BOCs simultaneously in every study.

Also, a particular concern is that no reviewed studies quantified bone stresses under the plate to assess bone “stress shielding” risk (i.e., BOC3), since the mismatch in mechanical properties between the cortical bone and metal plate could lead to bone density loss and plate loosening [13, 14]. Thus, future DHFP researchers should address bone “stress shielding” risk.

Furthermore, no studies considered the complete five-stage “lifecycle” (i.e., design, fabricate, inspect, repair, and dispose) for DHFPs, which is important for engineering products [99]. Such variability amongst DHFP biomechanical studies made interstudy comparisons challenging. Thus, future DHFP researchers could use standardized methodologies [100, 101], report the same outcomes [100, 101], and employ a “lifecycle” strategy to develop implants [99].

### 4.4. Clinical Recommendations

Clinical publications on DHFPs should be consulted to ensure that engineering optimization benefits patients. Plates implanted using the following principles can improve construct stability, fracture union rate, reoperation risk, postoperative function score, and/or elbow mobility [3, 102–104]: (i) plates should have the highest possible Os, Fs, and fatigue life; (ii) plates should give compression in the supracondylar zone for both humeral columns; (iii) parallel plates rather than perpendicular plates; (iv) locked rather than nonlocked plates, especially for low bone quality; and (v) plates should be as distal as possible without intruding into the joint space. Screws can improve construct stability as well as increase fracture union and full elbow mobility after surgery if these dictums are followed [102, 104, 105]: (i) screws should be as long as possible, (ii) screws should be as numerous as possible, (iii) each screw passes through as many bone fragments as possible, (iv) each screw's threads interdigitate with other screw's threads to create a fixed-angle construct, and (v) each screw passes through a plate hole.

## 5. Conclusion

This article summarized DHFP biomechanical design optimization publications that systematically evaluated plate and screw variables. This survey can assist engineers and surgeons in designing, analyzing, or utilizing DHFPs.

Firstly, there were 52 eligible studies that examined plate geometry, plate hole type, plate number, plate position, screw size, screw threads, screw number, screw distribution, and/or screw angle, but no studies evaluated alternative nonmetallic materials for plates or screws.

Secondly, studies used various combinations of biomechanical outcome metrics, such as interfragmentary fracture motion, bone, plate, or screw stress, number of loading cycles to failure, Os, and/or Fs, but no studies considered bone stress under the plate to lessen bone “stress shielding.”

Thirdly, the combined evidence from nine “key concepts” showed that a potentially ideal repair of an IAF or EAF of the distal humerus would use two precontoured, long, thick, locked, parallel metal plates that are affixed by long, thick, plate-to-plate metal screws that are positioned at staggered levels along the proximal sections of the plates, as well as an extra transfracture plate screw made from metal, but there was no definitive evidence for the best angle for the screws.

Finally, 20 practical recommendations were given for implant factors (e.g., future work needed on alternative nonmetallic materials, screw threads, and screw distribution), patient factors (e.g., future work needed on different bone densities, realistic fracture patterns, and multiple loading modes), study methodology (e.g., benefits of experimental validation of computational models, cadaveric rather than artificial humeri, and analyzing all four BOCs), and clinical aspects (e.g., benefits of locked plates, distally placed plates, and longer screws).

## Figures and Tables

**Table 1:** Biomechanical design optimization studies on DHFP variables for intraarticular fractures.

**Study characteristics**			**BOC1**	**BOC2**	**BOC3**	**BOC4**	**Other**
**Reference**	**Type**	**Variable**	**IFM**	**σ**	**σ**_**B** **U** **P**_	**Nf**	**Os, Fs**
Atalar et al. [32]	Exp, A, N, a, b	PP					●
Borbas et al. [33]	Exp, B, U, a	PG					●
Borbas et al. [34]	Exp, B, U, a	PG					●
Cai et al. [35]	Exp, B, U, a	PP		●			●
Got et al. [36]	Exp, B, O, b, t	PP					●
Hara et al. [37]	Exp, A, U, a	SN	●				●
Hungerer et al. [38]	Exp, A-N, B-U, a, b	PH, SN, SS	●			●	●
Jitprapaikulsarn et al. [39]	Fem, O, a, b, t	SA	●	●			
Kong et al. [40]	Fem, O, a, b, t	PP		●			●
Kruszewski et al. [41]	Fem, N, a, b	PP	●				
Kudo et al. [42]	Exp, A, U, a	PP	●				●
Penzkofer et al. [43]	Exp, A, N, a, b	PP	●				●
Schuster et al. [44]	Exp, B, O, a, b	PH				●	●
Schwartz et al. [45]	Exp, A, N, a, b, t	PP		●			●
Self et al. [46]	Exp, B, U, a	PP, ST					●
Shih et al. [47]	Exp, B, O, a, b	PG, PP	●				●
Shimamura et al. [48]	Exp, B, U, a, b	PN, SN					●
Stoffel et al. [49]	Exp, B, O, a, t	PP					●
Taylor et al. [50]	Exp, B, O, b, t	PP					●
Varady et al. [51]	Exp, A, N, a	PP, SA					●
Voigt et al. [52]	Exp, A, N, O, a, b	PG, PH	●			●	●
Wei, Ling, and An [53]	Fem, N, a, b, t	PP		●			●
Windolf et al. [54]	Exp, B, U, a, b	PP	●			●	●
Wright et al. [55]	Exp, B, U, a	PP	●				●
Zalavras et al. [56]	Exp, B, U, a, b	PP					●
Zha et al. [57]	Exp, A, N, a, b, t	PN, PP					●
Zhong et al.[58]	Fem, U, a, b, t	PG		●			

*Note:* Black circles (●) show which outcomes were measured or computed. Experiments were done using quasistatic and/or cyclic loads.

Abbreviations: *σ* = bone, plate, and/or screw peak stress; *σ*_BUP_ = bone stress under the plate; a, b, or t = axial, bending, or torsion; A = artificial humeri; B = biological humeri; BOC = biomechanical optimization criteria; Exp = experiments; Fem = finite element models; Fs = failure strength; IFM = interfragmentary motion reported as axial linear, shear linear, 3D linear, or “wedging” angular units; N, O, or U = normal, osteoporotic/osteopenic, or unknown bone quality; Nf = “fatigue life” defined as the number of loading cycles to failure; Os = overall stiffness; PG = plate geometry; PH = plate hole type; PN = plate number; PP = plate position; SA = screw angle; SN = screw number; SS = screw size; ST = screw threads.

**Table 2:** Biomechanical design optimization studies on DHFP variables for extraarticular fractures.

**Study characteristics**			**BOC1**	**BOC2**	**BOC3**	**BOC4**	**Other**
**Reference**	**Type**	**Variable**	**IFM**	**σ**	**σ**_**B** **U** **P**_	**Nf**	**Os, Fs**
Acar et al. [16]	Exp, A, N, a, b, t	PN, PP					●
Adamović et al. [59]	Exp, A, N, O, a, b	PG, PH	●				●
Arnander et al. [17]	Exp, A, U, b	PP					●
Bogataj et al. [60]	Fem, N, a, b, t	PP	●	●			
Caravaggi et al. [61]	Exp, B, N, O, a, b	PH, PP					●
Damron et al. [62]	Exp, B, U, t	PN					●
Filipowicz et al. [63]	Exp, B, U, a, t	PH					●
Hackl et al. [64]	Exp, B, U, b	PG, SD					●
Helfet and Hotchkiss [65]	Exp, B, U, b	PG				●	●
Hurt et al. [18]	Exp, B, U, a, t	PN					●
Jacobson, Glisson, and Urbaniak [66]	Exp, B, U, b, t	PN, PP					●
Jian-Qiao Peng et al. [67]	Fem, O, a, b, t	PP	●	●			
Kollias et al. [68]	Exp, B, U, b, t	PP				●	●
Koonce, Baldini, and Morgan [69]	Exp, B, O, a, b, t	PH, PP					●
Korner et al. [70]	Exp, B, U, a, b, t	PH, PP					●
Lim et al. [71]	Exp, B, U, a, b, t	PG					●
Mehling et al. [72]	Exp, B, U, a, b	SS					●
Mutlu et al. [73]	Exp, A, N, b, t	PP					●
Nourbakhsh et al. [74]	Exp, B, U, a	SA					●
Sabalic, Kodvanj, and Pavic [75]	Fem, O, a, b	PG, PP	●	●			●
Schemitsch, Tencer, and Henley [76]	Exp, B, U, a, b, t	PN, PP					●
Scolaro et al. [77]	Exp, A, U, b, t	PG, PN					●
Tejwani et al. [78]	Exp, B, U, a, b, t	PN	●				●
Thomrungpiyathan et al. [79]	Fem, N, V, a	PN	●	●			●
Zarifian et al. [80]	Fem, N, V, a, b, t	PG	●	●			●

*Note:* Black circles (●) show which outcomes were measured or computed. Experiments were done using quasistatic and/or cyclic loads.

Abbreviations: *σ* = bone, plate, and/or screw peak stress; *σ*_BUP_ = bone stress under the plate; a, b, or t = axial, bending, or torsion; A = artificial humeri; B = biological humeri; BOC = biomechanical optimization criteria; Exp = experiments; Fem = finite element models; Fs = failure strength; IFM = interfragmentary motion reported as axial linear, shear linear, 3D linear, or “wedging” angular units; N, O, or U = normal, osteoporotic/osteopenic, or unknown bone quality; Nf = “fatigue life” defined as the number of loading cycles to failure; Os = overall stiffness; PG = plate geometry; PH = plate hole type; PN = plate number; PP = plate position; SA = screw angle; SD = screw distribution; SS = screw size; V = validation of the finite element model using experiments.

**Table 3:** Ranges for combined data from all reviewed DHFP studies. The current authors did the following: multiplied the reported bone strains by an estimated elastic modulus to obtain bone stresses [35], resolved the reported axial+bending Os and Fs into axial and bending components [56], and used the reported lever arm lengths to convert bending Fs to N units [56] and bending Os to N/mm units [76].

**Outcome metrics**		**IAF**	**Ref.**	**EAF**	**Ref.**
BOC1	IFM: axial (mm)	0–4.60	[38, 41, 43, 47, 52, 54, 55]	0.01–1.30	[59, 78, 79]
	IFM: shear (mm)	0–2.22	[39, 41, 43]	—	—
	IFM: 3D (mm)	—	—	0–2.38	[60, 67, 75, 80]
	IFM: “wedging” (°)	0.006–5.40	[37, 42, 55]	—	—

BOC2	*σ*: bone (MPa)	0.13–936	[35, 39, 40, 53, 58]	4.92–25	[75, 80]
	*σ*: plate (MPa)	1.73–1285	[39, 40, 45, 53, 58]	10–1050	[60, 67, 75, 79, 80]
	*σ*: screws (MPa)	297–865	[53, 58]	—	—

BOC3	*σ*_BUP_ (MPa)	—	—	—	—

BOC4	Nf: axial (cycles)	2013–140,386	[38, 44, 54]	—	—
	Nf: bending (cycles)	13,000–57,000	[52]	223–5376	[65, 68]
	Nf: torsion (cycles)	—	—	—	—

Other	Os: axial (N/mm)	52–2456	[32–35, 37, 38, 40, 42–47, 49, 51–57]	34–4340	[16, 18, 59, 61, 63, 69–72, 74–76, 78–80]
	Os: bending (N/mm)	9–627	[32, 36, 38, 40, 43–45, 47, 50, 52, 54, 56, 57]	2–4500	[16, 17, 59, 61, 65, 68–73, 75–78, 80]
	Os: bending (Nm/°)	0–12.8	[53, 56]	—	—
	Os: torsion (Nm/°)	0.66–5.55	[40, 45, 49, 50, 53, 57]	0.14–40	[16, 18, 62, 63, 66, 68–71, 73, 76–78, 80]
	Fs: axial (N)	50–1796	[33, 34, 38, 43, 46–48, 56]	234–4406	[18, 63, 74]
	Fs: bending (N)	19–987	[32, 47, 48, 52, 56]	215–1618	[17, 61, 64, 69–71, 78]
	Fs: torsion (Nm)	14–44	[36, 49]	6.13–39	[62, 77]

Abbreviations: *σ*, peak stress; *σ*_BUP_, bone stress under the plate; BOC, biomechanical optimization criterion; EAF, extraarticular fracture; Fs, failure strength; IAF, intraarticular fracture; IFM, interfragmentary motion; Nf, number of loading cycles to failure; Os, overall stiffness.

**Table 4:** Design diversity of typical commercial DHFPs.

**Company**	**Plate name**	**Material**	**Length (mm)**	**Width (mm)**	**Thickness (mm)**	**Number of holes**
Medial plates						
7S Medical [85]	Distal Medial Humeral Plate	Titanium	84, 96, 108, 120, 132, 144, or 156	10	3.1	3, 4, 5, 6, 7, 8, or 9
Synthes [86]	Medial Distal Humerus Plates	Titanium or steel	58, 83, 110, 149, or 201	11	2.5	3, 5, 7, 9, or 14
TST [87]	Distal Humerus Medial Plates	Titanium or steel	98, 123, or 148	11.8	2	8, 10, or 12
Lateral plates						
7S Medical [85]	Distal Lateral Humeral Plate	Titanium	80, 92, 104, 116, 128, 140, or 152	10	3.1	3, 4, 5, 6, 7, 8, or 9
Synthes [86]	Posterolateral Distal Humerus Plates	Titanium or steel	55, 90, 116, 143, or 208	11	2.5	3, 5, 7, 9, or 14
TST [87]	Distal Humerus Lateral Plates	Titanium or steel	80, 92, or 105	11	4	8, 9, or 10

## Data Availability

The authors have nothing to report.
